# Supplementary material for: Enhanced genetic fine mapping accuracy with Bayesian Linear Regression models in diverse genetic architectures
Source: PLoS Genet. 2025 Jul 30;21(7):e1011783. doi: 10.1371/journal.pgen.1011783 (PMC12327644; doi:10.1371/journal.pgen.1011783)
Supplement: S1 Text — (DOCX) [file pgen.1011783.s001.docx]

**S1. Design of multiple credible sets in a fine-mapping region for the UKB phenotypes**

**Credible sets procedure for one causal SNPs (CS1)**

We define credible sets (CSs) as the smallest set of SNPs that include at least one causal variant, determined based only on PIPs (15).

1. SNPs in the fine mapping region were sorted by their PIP values in descending order.
2. A vector of cumulative sum of PIPs was created.
3. SNPs were included into the set until the cumulative sum of PIPs exceeded 99% probability.

If condition (3) was met, the fine mapping region would harbor one CS. The CS can contain one SNP to multiple SNPs depending on the cumulative sum of PIPs in the region.

In our simulations, we aimed to compare the core algorithms across different models by designing credible sets (CSs) uniformly, regardless of whether the models inherently produce CSs. For example, while FINEMAP-Inf does not generate CSs, we utilized its posterior inclusion probabilities (PIPs) to construct CSs. In cases where multiple SNPs had the same PIP value, we reviewed the list of SNPs and included the simulated causal SNP in the CS if it was among them. This approach was consistently applied across all models. Additionally, as we simulated only one causal SNP per fine-mapping region and did not consider overlaps between regions, the assumption of one causal SNP per region was preserved. This ensured the validity of our CS design.

***Credible set procedure for multiple causal SNPs (CS2)***

Fine-mapped regions in the additional simulations harbored two causal SNPs. To capture these causal SNPs we designed credible sets (CSs) based on PIP and LD. We used the following algorithm, as used by others (7, 24), to identify CS. Each SNP in the fine mapping region has obtained a PIP represented in a vector, $\boldsymbol{d}$.

Step 1. Sort the vector $\boldsymbol{d}$ in descending order, $\boldsymbol{d}_{sorted}$.

Step 2. Identify credible sets containing one SNP

- If any SNP in $\boldsymbol{d}_{sorted}$ has a PIP greater or equal to 0.90, it is defined as a credible set of size 1.
- This SNP (or multiple SNPs) is then removed from $\boldsymbol{d}_{sorted}$.

Step 3. Identify credible sets containing more than one SNP

- Starting from the remaining SNPs in $\boldsymbol{d}_{sorted}$, identify the SNP with the highest PIP, i.e., the lead variant, and variants with LD r^2^ >= 0.5.
- If the cumulative PIP of the lead variant and variants in strong LD is greater or equal to 0.90, these SNPs form a credible set and are removed from $\boldsymbol{d}_{sorted}$.

Step 4. Repeat steps under step 3 for each remaining genetic variant in $\boldsymbol{d}_{sorted}$.
