# Supplementary figures and images for: Enhanced genetic fine mapping accuracy with Bayesian Linear Regression models in diverse genetic architectures
Source: PLoS Genet. 2025 Jul 30;21(7):e1011783. doi: 10.1371/journal.pgen.1011783 (PMC12327644; doi:10.1371/journal.pgen.1011783)

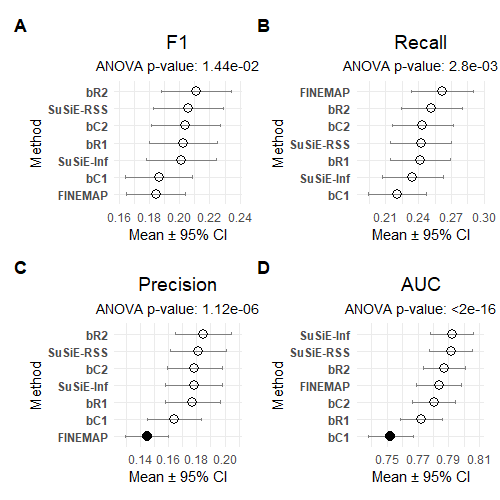

Supplement: S1 Fig — Forest plots display the mean performance (±95% confidence intervals) of each fine-mapping method across four metrics: F1-score (panel A), Recall (panel B), Precision (panel C), and AUC (panel D). The CS2 credible set procedure is used for the BLR models, while FINEMAP, SuSiE-RSS, and SuSiE-Inf use their respective internal procedures. Each panel shows the mean and 95% confidence interval of the specified metric across simulation replicates. Points are color-coded to indicate whether a method’s performance significantly differs (p < 0.05, based on a one-sample t-test) from the overall mean across all methods. Solid black points indicate statistically significant deviations; hollow circles indicate non-significant differences. Horizontal error bars represent the 95% confidence intervals. Methods are sorted by their mean score, with higher values indicating better performance. (TIF) [file pgen.1011783.s002.tif]

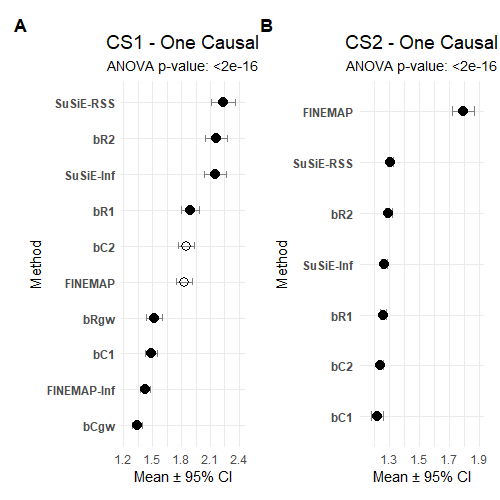

Supplement: S2 Fig — Forest plots show method-wise mean credible set size (±95% confidence intervals) across simulation replicates. Panel A shows results from one-causal regions using the CS1 credible set procedure applied to all methods. Panel B also reflects one-causal regions but uses the CS2 procedure for BLR models and the internal credible set procedures of FINEMAP, SuSiE-RSS, and SuSiE-Inf. Points are color-coded to indicate whether a method’s performance significantly differs (p < 0.05, based on a one-sample t-test) from the overall mean across methods. Solid black points indicate statistically significant deviations; hollow circles indicate non-significant differences. Horizontal error bars represent the 95% confidence intervals. Methods are sorted by their mean credible set size, with lower values indicating better performance. (TIF) [file pgen.1011783.s003.tif]

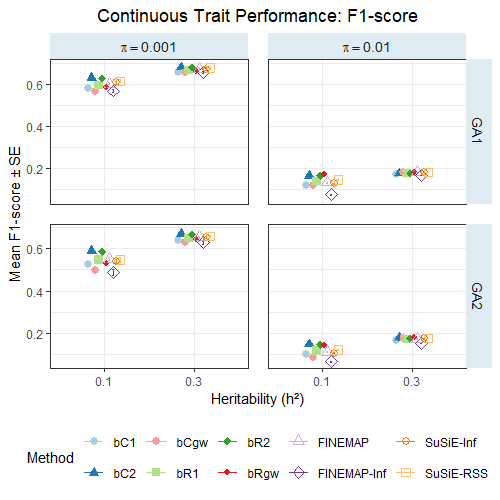

Supplement: S3 Fig — Results are stratified by heritability ($h^2$), proportion of causal variants ($\pi$), and genetic architecture (GA). Each panel represents a distinct value of $\pi$, with rows corresponding to genetic architecture (GA1 and GA2). Data points indicate the mean AUC performance (±SE) of each method across simulation replicates. Solid shapes denote the bC and bR methods; hollow shapes represent SuSiE and FINEMAP variants. Higher values indicate better performance. (TIF) [file pgen.1011783.s004.tif]

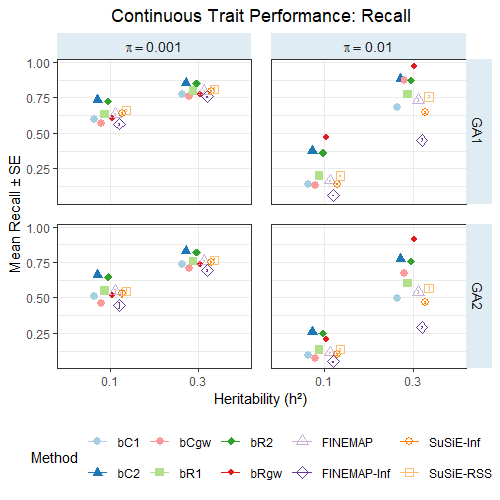

Supplement: S4 Fig — Results are stratified by heritability ($h^2$), proportion of causal variants ($\pi$), and genetic architecture (GA). Each panel represents a distinct value of $\pi$, with rows corresponding to genetic architecture (GA1 and GA2). Data points indicate the mean AUC performance (±SE) of each method across simulation replicates. Solid shapes denote the bC and bR methods; hollow shapes represent SuSiE and FINEMAP variants. Higher values indicate better performance. (TIF) [file pgen.1011783.s005.tif]

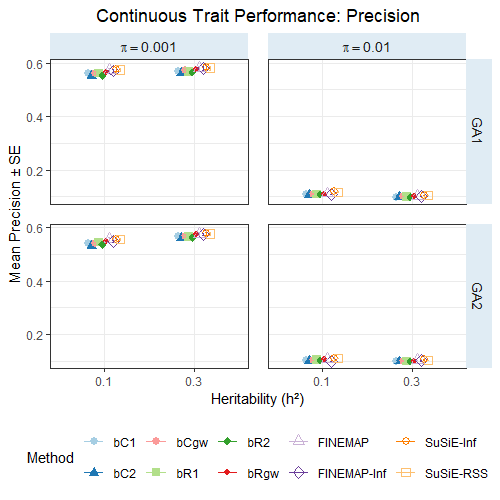

Supplement: S5 Fig — Results are stratified by heritability ($h^2$), proportion of causal variants ($\pi$), and genetic architecture (GA). Each panel represents a distinct value of $\pi$, with rows corresponding to genetic architecture (GA1 and GA2). Data points indicate the mean AUC performance (±SE) of each method across simulation replicates. Solid shapes denote the bC and bR methods; hollow shapes represent SuSiE and FINEMAP variants. Higher values indicate better performance. (TIF) [file pgen.1011783.s006.tif]

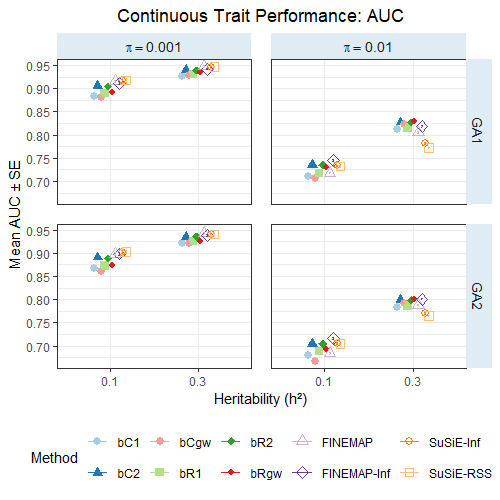

Supplement: S6 Fig — Results are stratified by heritability ($h^2$), proportion of causal variants ($\pi$), genetic architecture (GA), and disease prevalence ($p_v$). Each panel represents a distinct value of $\pi$, with rows corresponding to genetic architecture (GA1 and GA2). Data points indicate the mean AUC performance (±SE) of each method across simulation replicates. Solid shapes denote the bC and bR methods; hollow shapes represent SuSiE and FINEMAP variants. Higher values indicate better performance. (TIF) [file pgen.1011783.s007.tif]

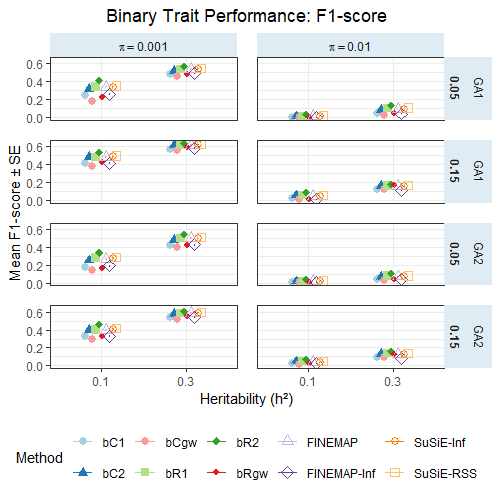

Supplement: S7 Fig — Results are stratified by heritability ($h^2$), proportion of causal variants ($\pi$), genetic architecture (GA), and disease prevalence ($p_v$). Each panel represents a distinct value of $\pi$, with rows corresponding to combinations of prevalence ($p_v$ = 0.05 or 0.15) and genetic architecture (GA1 or GA2). Data points indicate the mean AUC performance (±SE) of each method across simulation replicates. Solid shapes denote the bC and bR methods; hollow shapes represent SuSiE and FINEMAP variants. Higher values indicate better performance. (TIF) [file pgen.1011783.s008.tif]

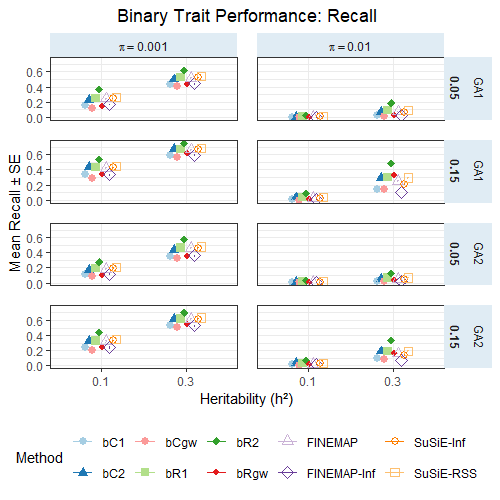

Supplement: S8 Fig — Results are stratified by heritability ($h^2$), proportion of causal variants ($\pi$), genetic architecture (GA), and disease prevalence ($p_v$). Each panel represents a distinct value of $\pi$, with rows corresponding to combinations of prevalence ($p_v$ = 0.05 or 0.15) and genetic architecture (GA1 or GA2). Data points indicate the mean AUC performance (±SE) of each method across simulation replicates. Solid shapes denote the bC and bR methods; hollow shapes represent SuSiE and FINEMAP variants. Higher values indicate better performance. (TIF) [file pgen.1011783.s009.tif]

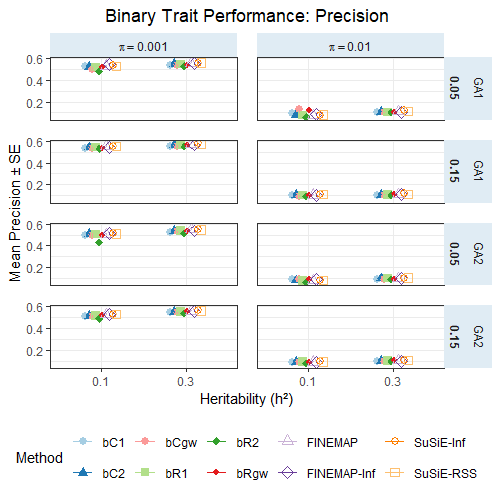

Supplement: S9 Fig — Results are stratified by heritability ($h^2$), proportion of causal variants ($\pi$), genetic architecture (GA), and disease prevalence ($p_v$). Each panel represents a distinct value of $\pi$, with rows corresponding to combinations of prevalence ($p_v$ = 0.05 or 0.15) and genetic architecture (GA1 or GA2). Data points indicate the mean AUC performance (±SE) of each method across simulation replicates. Solid shapes denote the bC and bR methods; hollow shapes represent SuSiE and FINEMAP variants. Higher values indicate better performance. (TIF) [file pgen.1011783.s010.tif]

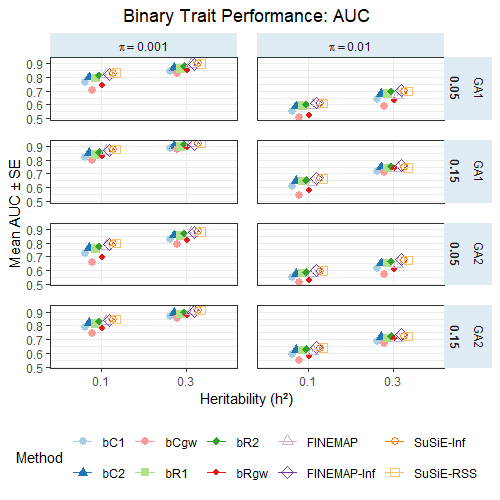

Supplement: S10 Fig — Results are stratified by heritability ($h^2$), proportion of causal variants ($\pi$), genetic architecture (GA), and disease prevalence ($p_v$). Each panel represents a distinct value of $\pi$, with rows corresponding to combinations of prevalence ($p_v$ = 0.05 or 0.15) and genetic architecture (GA1 or GA2). Data points indicate the mean AUC performance (±SE) of each method across simulation replicates. Solid shapes denote the bC and bR methods; hollow shapes represent SuSiE and FINEMAP variants. Higher values indicate better performance. (TIF) [file pgen.1011783.s011.tif]

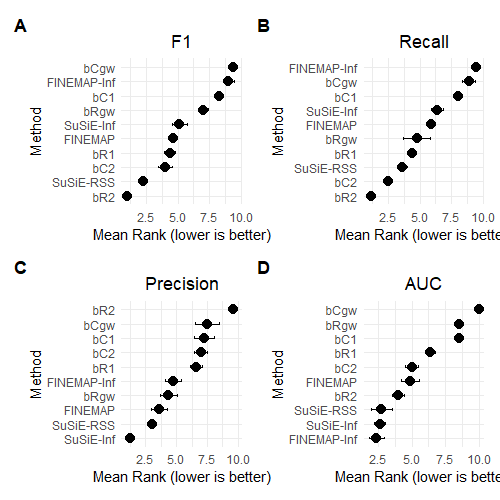

Supplement: S11 Fig — Mean rank ± standard error (SE) of method performance across simulation settings for four evaluation metrics using credible set method CS1 (simple approach). This multi-panel figure shows the mean rank ± standard error (SE) for each fine-mapping method based on four metrics: F1-score (panel A), Recall (panel B), Precision (panel C), and AUC (panel D). For each metric, methods were ranked within each simulation setting defined by combinations of genetic architecture (GA), polygenicity ($\pi$), and heritability ($h^2$). Lower ranks indicate better performance. The ranks were then averaged across all settings, and the standard error was computed to reflect variability in ranks. Each point in the plot represents the average rank of a method, with error bars indicating ±1 SE. Methods that consistently rank higher across simulations appear lower on the vertical axis. This visualization allows a direct comparison of method stability and performance across multiple evaluation criteria. (TIF) [file pgen.1011783.s012.tif]

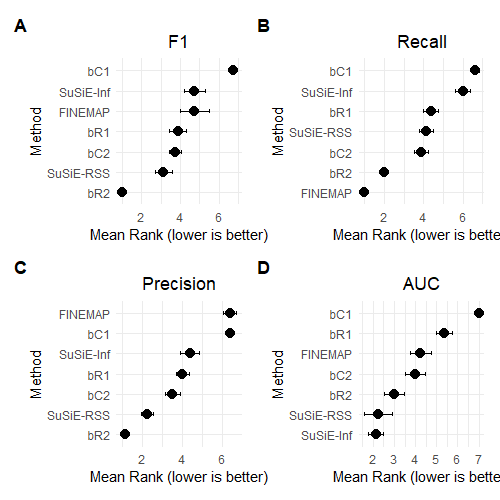

Supplement: S12 Fig — Mean rank ± standard error (SE) of method performance across simulation settings for four evaluation metrics using credible set method CS2 (advanced approach).This multi-panel figure shows the mean rank ± standard error (SE) for each fine-mapping method based on four metrics: F1-score (panel A), Recall (panel B), Precision (panel C), and AUC (panel D). For each metric, methods were ranked within each simulation setting defined by combinations of genetic architecture (GA), polygenicity ($\pi$), and heritability ($h^2$). Lower ranks indicate better performance. The ranks were then averaged across all settings, and the standard error was computed to reflect variability in ranks. Each point in the plot represents the average rank of a method, with error bars indicating ±1 SE. Methods that consistently rank higher across simulations appear lower on the vertical axis. This visualization allows a direct comparison of method stability and performance across multiple evaluation criteria. (TIF) [file pgen.1011783.s013.tif]

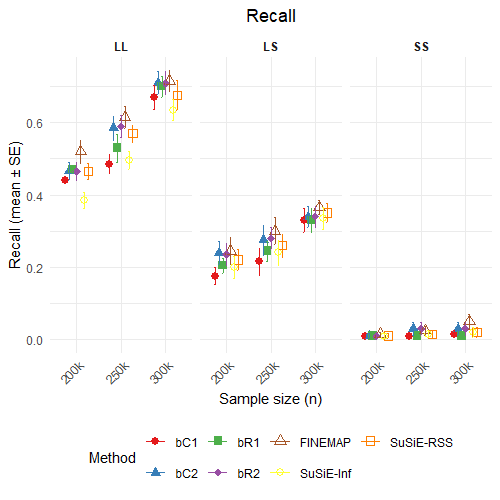

Supplement: S13 Fig — Each point represents the mean Recall of a method (indicated by color and shape) across simulation replicates for a given sample size (n = 200k, 250k, 300k) and causal configuration. Causal configurations are grouped as LL (two large-effect causal variants), LS (one large and one small effect), and SS (two small-effect variants). Error bars indicate ±1 standard error. Higher values indicate better performance. (TIF) [file pgen.1011783.s014.tif]

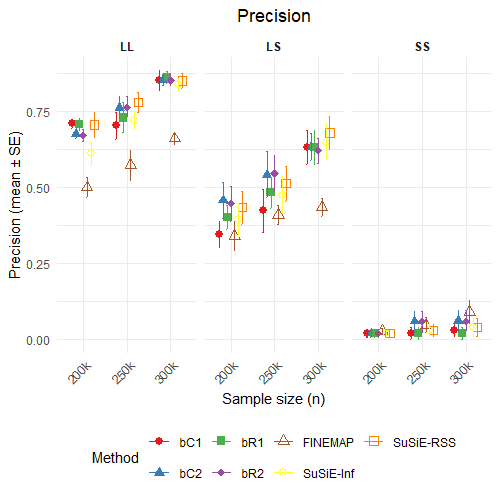

Supplement: S14 Fig — Each point represents the mean Recall of a method (indicated by color and shape) across simulation replicates for a given sample size (n = 200k, 250k, 300k) and causal configuration. Causal configurations are grouped as LL (two large-effect causal variants), LS (one large and one small effect), and SS (two small-effect variants). Error bars indicate ±1 standard error. Higher values indicate better performance. (TIF) [file pgen.1011783.s015.tif]

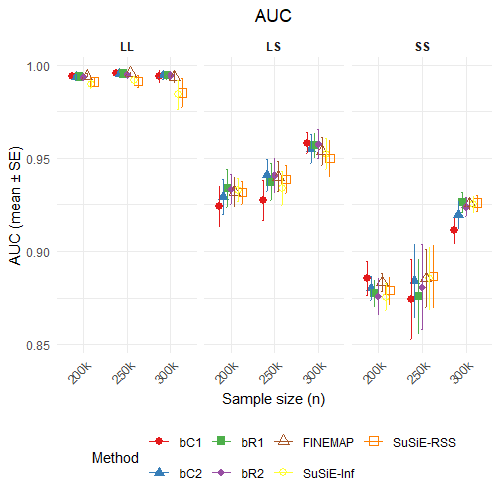

Supplement: S15 Fig — Each point represents the mean Recall of a method (indicated by color and shape) across simulation replicates for a given sample size (n = 200k, 250k, 300k) and causal configuration. Causal configurations are grouped as LL (two large-effect causal variants), LS (one large and one small effect), and SS (two small-effect variants). Error bars indicate ±1 standard error. Higher values indicate better performance. (TIF) [file pgen.1011783.s016.tif]

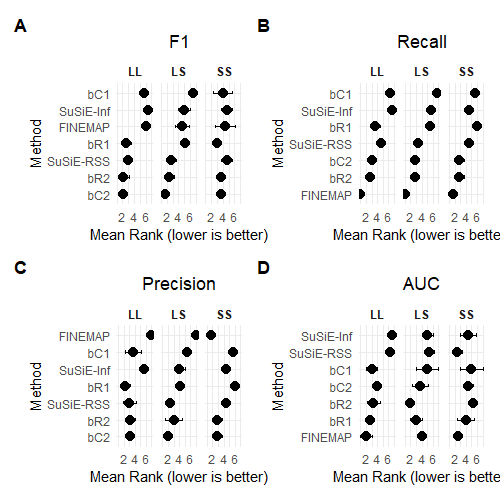

Supplement: S16 Fig — This multi-panel figure presents the mean performance rank (± standard error) of each fine-mapping method across four evaluation metrics: F1-score (panel A), Recall (panel B), Precision (panel C), and AUC (panel D). For each metric, methods were ranked within each combination of sample size and causal configuration, with lower ranks indicating better performance. The figure shows the average rank and corresponding standard error across all sample sizes, with panels faceted by causal architecture. Causal configurations are grouped as LL (two large-effect causal variants), LS (one large- and one small-effect variant), and SS (two small-effect variants). Error bars indicate ±1 standard error. Lower mean ranks reflect better overall performance. (TIF) [file pgen.1011783.s017.tif]

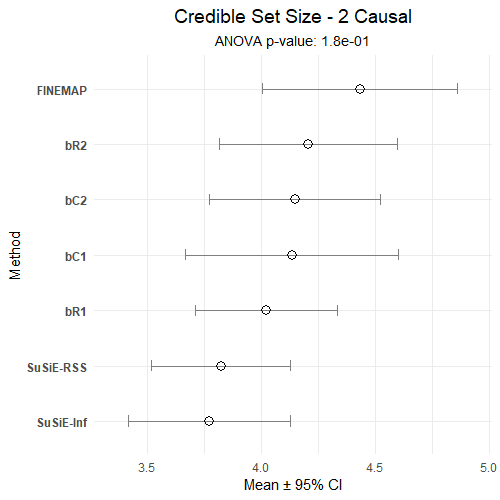

Supplement: S17 Fig — Forest plots show method-wise mean credible set size (±95% confidence intervals) across simulation replicates. The CS2 credible set procedure for the BLR models and the internal credible set procedures implemented in FINEMAP, SuSiE-RSS, and SuSiE-Inf. Points are color-coded to indicate whether a method’s performance significantly differs (p < 0.05, based on a one-sample t-test) from the overall mean across all methods. Solid black points indicate statistically significant deviations; hollow circles indicate non-significant differences. Horizontal error bars represent the 95% confidence intervals. Methods are sorted by their mean score, with lower values indicating better performance. (TIF) [file pgen.1011783.s018.tif]

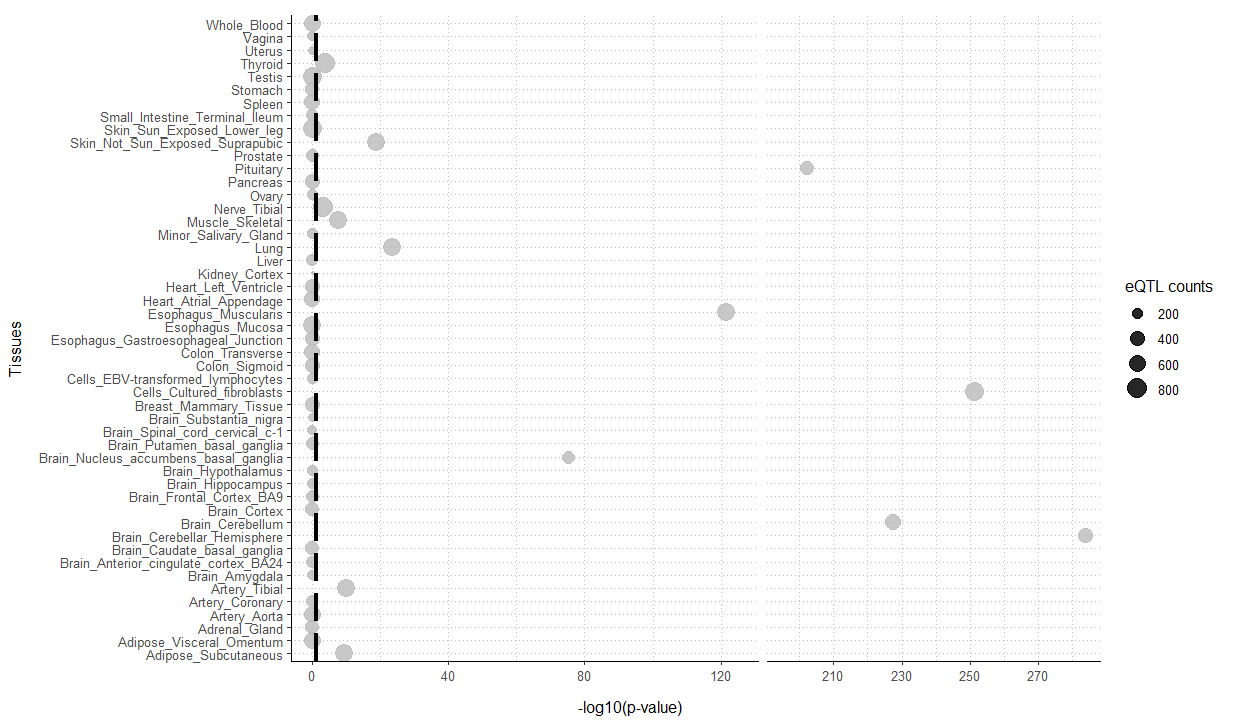

Supplement: S18 Fig — The black dashed line represents the significance cut-off (p-value < 0.05). The size of the points corresponds to the number of eQTLs (eQTL counts) in the tissue. (PNG) [file pgen.1011783.s019.png]
