# Supplementary material for: Enhanced genetic fine mapping accuracy with Bayesian Linear Regression models in diverse genetic architectures
Source: PLoS Genet. 2025 Jul 30;21(7):e1011783. doi: 10.1371/journal.pgen.1011783 (PMC12327644; doi:10.1371/journal.pgen.1011783)
Supplement: Attachment — Submitted filename: Reviewer_Comments_GENETICS-D-24-00378.pdf [file pgen.1011783.s027.pdf]

Title: Evaluation of Bayesian Linear Regression Models as a Fine Mapping tool
Manuscript ID: PGENETICS-D-24-00378

In this manuscript, the authors proposed the fine-mapping tool based on the Bayesian Linear Regression (BLR) models with BayesC and BayesR priors. The article is well-written, clear, and easy to follow. The content should be interesting to readers. While the methods looks reasonable, I have several concerns and comments:

Main comments

1. In the abstract, the authors concluded that 'BayesR improved the F1 score, averaged across all simulations, between 27.26% and 13.32% relative to the external models.' However, in Figure 5, the precision (TP/(TP+FN)) of bR3 and bR2 is much smaller than that of other methods. Additionally, bR1 only has the same performance as SuSiE-RSS and SuSiE-Inf. It would be beneficial to include more discussions on these points to provide a balanced view. Otherwise, the abstract conclusion may mislead users into favoring the BayesR prior in the BLR model without fully understanding its comparative performance.

2. It is not clear how the credible sets are constructed, as they are defined differently for simulations and real data analysis. More details are needed to understand the criteria and methods used for defining credible sets in both contexts to ensure consistency and clarity for the reader. The main concern is when the authors merged the fine-mapping regions in the real data analysis, multiple credible sets scenarios will be very common in general, and this is the most important part to pinpointing the results from the method the authors proposed in this manuscript (only shown in Figure S1 and Text S1 in current version).

3. What happens if there are two causal variants in the same region with moderate LD (e.g., LD $\sim$ 0.7)? It is important to understand how the model handles such scenarios, as moderate linkage disequilibrium can complicate the identification and interpretation of causal variants. Additional discussion on the model's performance and potential challenges in this situation would provide valuable insights.

4. It is not clear that 'BLR models' are introduced after the section 'Estimation of parameters using BLR models'. To improve clarity, it would be better to reorganize these two subsections by linking the three options of BLR models directly with the parameter estimation process. Otherwise, readers might lose insights into the meanings of bR1-3 and bC1-3 in Figure 5.

5. In the real data analysis of the UKB phenotypes, the authors divided the data into five replicates of training (80%) and validation (20%) populations. In this context, I am curious whether the authors used the same covariates based on the entire population or calculated the covariates separately for the training and validation populations.

6. Lines 361-367: It is not clear to the reader what is meant by genome-wide analysis based on the BayesR with Option 1 (bRgw). Additionally, using only one-tenth of the samples to estimate sparse LD for a group of SNPs may introduce bias in the LD estimation, potentially causing a mismatch with the GWAS summary statistics. How did the authors address this issue to ensure the accuracy of the LD estimates?

Corrections/minor comments

1. Lines 172, 184, and 247, it is better if the authors could use the consistent annotation for the centered and standardized genotype matrix.

2. Line 226, you added age, sex and UKB assessment center as covariates in the genetic analyses. Did you also include top PCs? Based on the line 324, I believed the authors also included top ten PCs as the covariates.

3. Some potential typos:

   - Line 115, missing a subject before "developed a standard..."
   - Line 257, it is better if the author could provide the detail explanation of σ_b^2 right after.
   - Line 309, "pi" should be π in math.
   - Line 415, "SNPS" should be "SNPs".

4. What is the computational time of the BLR models based on two different priors? Additionally, it is benifical to reader if the authors could provide summary of the set size (SNP counts in the CS), and the minimum correlation among SNPs in the CS for each methods.

5. Figure S5a and S5b, it is better to provide the total number of SNPs for each method under the method name.

6. The BLR model is based on a simple linear relationship between y and X for quantitative phenotypes. For binary phenotypes, a logistic regression model may be more appropriate. Did the authors compare the performance of the BLR model using a simple linear model for quantitative phenotypes and logistic regression for binary phenotypes? Including a comparison would provide a clearer understanding of the model's applicability and performance across different types of phenotypic data.
