# Supplementary material for: Enhanced genetic fine mapping accuracy with Bayesian Linear Regression models in diverse genetic architectures
Source: PLoS Genet. 2025 Jul 30;21(7):e1011783. doi: 10.1371/journal.pgen.1011783 (PMC12327644; doi:10.1371/journal.pgen.1011783)
Supplement: Attachment — Submitted filename: Response_to_reviewers.docx [file pgen.1011783.s029.docx]

Dear editor,

We are pleased to resubmit our manuscript titled “***Evaluation of Bayesian Linear Regression Models as a Fine Mapping tool***” (Manuscript ID: PGENETICS-D-24-00378) for consideration in PLOS Genetics. We sincerely appreciate the thorough feedback and constructive comments provided by the reviewers. These insights have been invaluable in strengthening our work, and we have made significant revisions to address all concerns raised during the review process.

In this revised version, we have made a great effort re-structuring the Method section, provide clearer definitions of e.g., how we define credible sets (which is similar to how it is defined by recently published methods; e.g., PMID: 35853082 and doi:10.1101/2024.07.18.24310667), and incorporated an entirely new section on simulations that shows the performance of the fine mapping methodology when there are more than one causal genetic variant in a region. In particular the latter point, has in our opinion, raised the quality of the entire manuscript. Based on comments, we have reconsidered our approach and decided to remove the results for the methods BayesR region-wide (bR3) and BayesC region-wide (bC3) as these methods produce the same outcomes as bR2 and bC2, respectively. A detailed point-by-point response to the reviewers' comments is included, outlining how each suggestion has been addressed.

We hope that the revisions meet the expectations of the reviewers and that the manuscript is now suitable for publication in PLOS Genetics.

Thank you for your time and consideration. We look forward to your feedback.

Sincerely, and on behalf of all coauthors,

Merina Shrestha

Postdoctoral researcher, PhD

Centre for Quantitative Genetics and Genomics, Aarhus University, Aarhus, Denmark

**Reviewer #1**

In this manuscript, the authors proposed the fine-mapping tool based on the Bayesian Linear Regression (BLR) models with BayesC and BayesR priors. The article is well-written, clear, and easy to follow. The content should be interesting to readers. While the methods looks reasonable, I have several concerns and comments:

***Main comments***

1. In the abstract, the authors concluded that ’BayesR improved the F1 score, averaged across all simulations, between 27.26% and 13.32% relative to the external models.’ However, in Figure 5, the precision (TP/(TP+FN)) of bR3 and bR2 is much smaller than that of other methods. Additionally, bR1 only has the same performance as SuSiE-RSS and SuSiE-Inf. It would be beneficial to include more discussions on these points to provide a balanced view. Otherwise, the abstract conclusion may mislead users into favoring the BayesR prior in the BLR model without fully understanding its comparative performance.

*Response-1*: Thank you for the comment. Based on the reviewers' feedback, we have removed the results for bR3 and retained only the results for bR2. The overall F1 score for bR2 is higher compared to other fine-mapping models. However, its precision remains lower than that of other models. To simplify the discussion, we have analyzed this issue within the context of BayesR priors by comparing bR1 and bR2. Additionally, we have explained why this pattern is not observed with BayesC priors. In brief, we believe the lower precision is related to the default parameter settings for the metric π in the BayesR prior. This issue is discussed in detail in the "F1 Classification Score – Recall and Precision of the BLR Models" section (see lines 800–817). For comparisons between bR2 and external fine-mapping models, we focused on recall, incorporating a penalty for large credible set sizes that can lead to lower recall estimates. This topic, along with related considerations, is discussed in the "Comparison of the BLR Models to External Models" section (see lines 819–839).

2. It is not clear how the credible sets are constructed, as they are defined differently for simulations and real data analysis. More details are needed to understand the criteria and methods used for defining credible sets in both contexts to ensure consistency and clarity for the reader. The main concern is when the authors merged the fine-mapping regions in the real data analysis, multiple credible sets scenarios will be very common in general, and this is the most important part to pinpointing the results from the method the authors proposed in this manuscript (only shown in Figure S1 and Text S1 in current version).

*Response-2*: It is accurate that two slightly different approaches were employed to define credible sets (CS) in the simulations and real data analysis. The primary distinction lies in whether linkage disequilibrium was taken into consideration. For the simulations, the approach outlined by Maller et al. (PMID: 23104008) was used, whereas for the real data analysis, the definition follows a methodology similar to that recently proposed by Zho et al. (PMID: 35853082) and Wu et al. (doi: 10.1101/2024.07.18.24310667). In the revised manuscript, we have worked on making a clearer description of the CS definitions (section “Credible sets for simulations”: lines 403-411, and section “Credible sets for simulations with multiple causal SNPs”: lines 423-442, and Supplementary Text: S1Text) including a flowchart describing the process (see Supplementary Figure: S1Fig.).

The approach outlined by Maller et al. (PMID: 23104008) assuming only one causal variant in a region creates only one credible set per fine-mapping region based only on PIPs of SNPs.

In our simulations, we designed a fine-mapping region based on one simulated causal SNP/variant. We didn’t combine regions irrespective of potential overlap of SNPs among fine-mapping regions. This holds our assumption of one simulated causal SNP in the region and use of the approach outlined by Maller et al. (PMID: 23104008).

For the real-data analysis, we designed a fine-mapping region based on significant SNPs from GWAS. First, the number of causal SNPs in a region is unknown. Moreover, we merged regions if the SNPs overlapped increasing chances of multiple causal SNPs in the region. Hence, we used an approach based on PIPs and LD information of the SNPs to obtain multiple credible sets per fine-mapping region. The same credible set design was also applied in an additional simulation we conducted, focusing on fine-mapping regions harbouring two causal genetic variants.

3. What happens if there are two causal variants in the same region with moderate LD (e.g., LD ∼ 0.7)? It is important to understand how the model handles such scenarios, as moderate linkage disequilibrium can complicate the identification and interpretation of causal variants. Additional discussion on the model’s performance and potential challenges in this situation would provide valuable insights.

*Response-3*: We acknowledge this important comment. Therefore, we have performed additional analyses simulating regions with two causal SNPs at varying levels of correlations between the two SNPs (please see Method description section “Two causal genetic variants” lines 182-189, Supplementary Table: S2 Table). Our additional simulations recapitulate the results already presented further highlighting the competitiveness of our methodology (please see description of results section “Comparison of fine-mapping models in “*Two causal genetic variants*”” lines 647-670).

4. It is not clear that ’BLR models’ are introduced after the section ’Estimation of parameters using BLR models’. To improve clarity, it would be better to reorganize these two subsections by linking the three options of BLR models directly with the parameter estimation process. Otherwise, readers might lose insights into the meanings of bR1-3 and bC1-3 in Figure.

*Response-4*: Thank you for the comment. In the revised version, we have made significant efforts to restructure the Methods section. We updated the title to better reflect the content and reorganized sections within the Methods section to enhance coherence. We hope these changes improve clarity and simplicity of our revised version.

5. In the real data analysis of the UKB phenotypes, the authors divided the data into five replicates of training (80%) and validation (20%) populations. In this context, I am curious whether the authors used the same covariates based on the entire population or calculated the covariates separately for the training and validation populations.

*Response-5*: The same set of covariates was applied to the entire population in the association analysis. Initially, we used a linear model for quantitative traits (or a logistic model for binary traits) to adjust for the effects of the covariates. The residuals from these models were then standardized and used in the single-marker regression analysis. This has been clarified in the revised manuscript (see lines 234-236).

6. Lines 361-367: It is not clear to the reader what is meant by genome-wide analysis based on the BayesR with Option 1 (bRgw). Additionally, using only one-tenth of the samples to estimate sparse LD for a group of SNPs may introduce bias in the LD estimation, potentially causing a mismatch with the GWAS summary statistics. How did the authors address this issue to ensure the accuracy of the LD estimates?

*Response-6*: Our procedure is similar to a genome-wide fine mapping procedure suggested by Wu et al. (PMID: 39072021). Current methods typically focus on individual genomic segments without considering the global genetic architecture. The paper by Wu et al. demonstrated the advantages of performing genome-wide fine-mapping including error control, mapping power and precision, replication rate, and trans-ancestry phenotype prediction.

We used 50,000 random samples from the UK Biobank as the in-sample LD reference to perform the fine-mapping analysis. This approach was based on the recommendations provided in the SBayesR paper by Lloyd et al. (PMID:31704910). Furthermore, we monitored the convergence of model parameters using the Geweke convergence diagnostic for Markov chains (https://doi.org/10.1093/oso/9780198522669.003.0010). This diagnostic tests whether the means of the first and last segments of the chain (default settings: the first 10% and the last 50%) are equal. The test statistic is a Z-score calculated as the difference between the two sample means divided by its estimated standard error. A threshold of 3.0 (i.e.,(absolute value of Z)>3) was used to detect non-convergence. In our experience, these diagnostics provide a simple and robust approach to assess whether the analysis has converged. For all analyses performed using our Bayesian linear regression (BLR) model framework with in-sample LD, we observed convergence. However, when using external LD references, such as the 1000 Genomes data derived from small sample sizes and meta-analysed GWAS summary statistics, we suggest to use a low-rank version of our BLR models as proposed by Wu et al. (PMID: 39072021) and Zheng et al. (PMID: 38689000). Convergence was monitored using the Geweke diagnostic in these cases as well. This procedure appears robust and is further detailed in the study by Zheng et al. (PMID: 38689000).

*Corrections/minor comments*

1. Lines 172, 184, and 247, it is better if the authors could use the consistent annotation for the centered and standardized genotype matrix.

*Response-1*: Thank you for pointing this out. The annotation for the centered and standardized genotype has been made standardized.

2. Line 226, you added age, sex and UKB assessment center as covariates in the genetic analyses. Did you also include top PCs? Based on the line 324, I believed the authors also included top ten PCs as the covariates.

*Response-2*: Yes, we included the top 10 PCs as co-variates along with age, sex and UKB assessment center in the genetic analysis. This has been clarified in the revised manuscript (see line 234-236).

3. Some potential typos:

- Line 115, missing a subject before ”developed a standard...”

- Line 257, it is better if the author could provide the detail explanation of σ2 b right after.

- Line 309, ”pi” should be π in math.

- Line 415, ”SNPS” should be ”SNPs”.

*Response-3*: Thank you very much for pointing us to these errors. They have all been fixed.

4. What is the computational time of the BLR models based on two different priors? Additionally, it is beneficial to reader if the authors could provide summary of the set size (SNP counts in the CS), and the minimum correlation among SNPs in the CS for each method.

*Response-4*: Table 2 in the manuscript contains information from the real trait analysis regarding number, median size, median LD among SNPs in the CSs for each method used.

For one scenario (h^2^ = 0.3, $\pi$ = 0.01 and genetic architecture GA1) we compared the computational time (in seconds) for BayesC and BayesR. The table below shows computation running time that involves both computation of LD and fine mapping within a region of 1001 SNPs.

| **Replicate** | **BayesC** | | **BayesR** | |
| --- | --- | --- | --- | --- |
|  | **opt1** | **opt2** | **opt1** | **opt2** |
| 1 | 1.09 | 1.05 | 1.45 | 1.44 |
| 2 | 1.09 | 1.06 | 1.45 | 1.43 |
| 3 | 0.90 | 0.86 | 1.22 | 1.22 |
| 4 | 0.89 | 0.87 | 1.34 | 1.35 |
| 5 | 1.12 | 1.11 | 1.46 | 1.48 |
| 6 | 1.10 | 1.09 | 1.46 | 1.49 |
| 7 | 1.04 | 1.03 | 1.33 | 1.35 |
| 8 | 0.89 | 0.82 | 1.37 | 1.38 |
| 9 | 1.08 | 1.05 | 1.41 | 1.42 |
| 10 | 1.13 | 1.10 | 1.48 | 1.48 |
| **Average** | **1.03** | **1.00** | **1.40** | **1.40** |

5. Figure S5a and S5b, it is better to provide the total number of SNPs for each method under the method name.

*Response-5*: The legend for Figure S5a and S5b has been rephrased, and now the total number of true positive for each method has been included under each method in the figure.

6. The BLR model is based on a simple linear relationship between y and X for quantitative phenotypes. For binary phenotypes, a logistic regression model may be more appropriate. Did the authors compare the performance of the BLR model using a simple linear model for quantitative phenotypes and logistic regression for binary phenotypes? Including a comparison would provide a clearer understanding of the model’s applicability and performance across different types of phenotypic data.

*Response-6*: The BLR model does not rely on individual-level data (phenotypes and genotypes); instead, it utilizes GWAS summary-level data. Estimating SNP effects ($\hat{\boldsymbol{b}}$) typically requires $\boldsymbol{X}’\boldsymbol{X}$ and $\boldsymbol{X}’\boldsymbol{y}$ (where $\boldsymbol{X}$ is the genotypes and $\boldsymbol{y}$ is the phenotypes). However, when these are unavailable, they, that is $\boldsymbol{X}’\boldsymbol{X}$ and $\boldsymbol{X}’\boldsymbol{y}$**,** can be reconstructed using an LD correlation matrix ($\boldsymbol{B}$) and publicly available GWAS summary statistics, as detailed in the manuscript (please see lines 253-268). In our study, we exclusively used GWAS summary data. For the UKB GWAS analyses, we conducted linear regression for quantitative traits and logistic regression for binary traits (implemented in PLINK), as described in detail in lines 232-238 of the manuscript.

**Reviewer #2**

This was an interesting manuscript that will be of interest/relevance to others working in the field. I have a few minor comments and two major comments.

***Major Comments***

1. Much of the interest in fine mapping relates to the situation where there is more than one causal variant in a region. Indeed, the authors explore this a bit in their UKB analyses. In the simulations, it is unclear to me whether many/any of the regions that ended up being fine mapped will have included more than one causal variant? Given the simulation parameters where \pi, the proportion of causal SNPs, is set to either 0.1% or 1%, it seems to me rather unlikely that any of the fine-mapped regions will have ended up harbouring more than one causal variant.

The authors need to include some simulation scenarios where they enforce a situation whereby there are multiple causal variants in a fine-mapped region. They then need took at the performance of the various methods *separately* in this situation (for comparison with the performance of the methods when there is only one causal variant per region).

*Response-1*: In our simulation analysis, we randomly selected causal SNPs genome-wide and defined fine-mapping regions around each causal SNP by including ±500 SNPs.

In the revised manuscript, we have performed additional simulations for scenarios with regions that contain two causal SNPs with varying degrees of LD between the two causal SNPs (please see Method section “Two causal genetic variants”: lines 182-189, Supplementary Table: S2 Table). Multiple credible sets were generated for all fine-mapping methods, and their ability to identify zero, one, or both causal SNPs was compared (please see description of results at lines 647-670, Supplementary Table: S4 Table).

2. The authors point out that they do not make any examination of the CSs that are actually output by FINEMAP, SuSIE-RSS and SuSIE-Inf - instead they define CSs using their own algorithm (essentially the same algorithm that they use for defining CSs from the BLR methods). Their justification for this is "the main objective of our study was to compare the efficiency of the algorithms of these models. Introducing a comparison based on the CSs they determine would introduce additional complexity and divert us far from our objective".

Unfortunately, I do not consider this satisfactory. I consider it ESSENTIAL that the authors include in their comparison the CSs that are actually output by FINEMAP, SuSIE-RSS and SuSIE-Inf. The authors of these software packages (particularly for the SuSIE methods) worked very hard to come up with a principled way of defining CSs, indeed this was possibly the most attractive and compelling feature of the original SuSIE method when it was introduced. In any case this default option is what people will be using in practice. I have no objection to the authors additionally including their own definition CSs in the comparison, but the more interesting comparison is with the default outputs.

*Response-2*: Thank you for your feedback and for highlighting the importance of including the default credible sets (CSs) output by tools such as FINEMAP, SuSiE-RSS, and SuSiE-Inf in our comparisons. We fully agree that the default CS definitions are an integral aspect of these methods and a key feature that practitioners rely on.

For our analyses, two slightly different approaches were used to define credible sets (CSs) in the simulations and real data analyses, with the main difference being whether linkage disequilibrium was taken into account. For the simulations, we adopted the method described by Maller et al. (PMID: 23104008), while for the real data analysis and the new added simulation, we utilized a definition aligned with the methodologies recently proposed by Zho et al. (PMID: 35853082) and Wu et al. (doi: 10.1101/2024.07.18.24310667). In our newly added simulation analyses, we compared the outputs of these fine-mapping tools with our BLR methods, explicitly including both their default CS definitions and the CSs derived from the BLR methods. This dual comparison allowed us to assess not only the default performance of these tools but also how their results align with the CSs defined using our approach.

We believe this comprehensive comparison provides a fair and balanced evaluation. It acknowledges the foundational work done by the authors of these tools in defining their CSs while also showcasing how the BLR-derived CSs compare under the same conditions. Our findings indicate that this dual perspective is valuable for understanding the relative strengths of each method and how they can be applied effectively in practice.

***Minor Comments***

1. The Abstract goes into too much nitty gritty of detailed results (including percentages etc.) and as a result is quite confusing. It needs to be more succinct, with a focus on summarising the main findings.

*Response-1*: We have rewritten the abstract neglecting the detailed results.

2. Page 6 line 130: "Type two diabetes" should probably read "Type 2 diabetes".

*Response-2*: Thank you for pointing this out. This has been corrected.

3. Page 7 line 149: You mention UKB but have you correctly acknowledged UKB in the manuscript? At <https://www.ukbiobank.ac.uk/enable-your-research/manage-your-project>
It says: Please ensure that you use the following in the Acknowledgments section in your paper:
"This research has been conducted using the UK Biobank Resource under Application Number xxxxx."

*Response-3*: This is now included under the Acknowledgement.

4. Page 9 line 192: Can you confirm that you will have ended up choosing different causal SNPs in each replicate of each simulation scenario?

*Response-4*: The causal SNPs for each replicate within a scenario were sampled at random, thus, it is highly unlikely that the same SNP was selected multiple time, and even if it was, the simulated SNP effect would not be the same (please see addition in revised manuscript lines 174-177). The table below summarises the overlap of SNPs within simulation scenarios across the ten independent replicates.

| **Simulation scenario** | **Total no. SNPs** | **Unique no. SNPs** | **No. SNPs sampled multiple times** | **% SNPs sampled multiple times** |
| --- | --- | --- | --- | --- |
| h2:0.1; snp.cau:0.001; ga:1 | 5330 | 5311 | 19 | 0.36 |
| h2:0.1; snp.cau:0.001; ga:2 | 5340 | 5309 | 31 | 0.58 |
| h2:0.1; snp.cau:0.01; ga:1 | 53360 | 50951 | 2409 | 4.51 |
| h2:0.1; snp.cau:0.01; ga:2 | 53370 | 51038 | 2332 | 4.37 |
| h2:0.3; snp.cau:0.001; ga:1 | 5330 | 5296 | 34 | 0.64 |
| h2:0.3; snp.cau:0.001; ga:2 | 5340 | 5312 | 28 | 0.52 |
| h2:0.3; snp.cau:0.01; ga:1 | 53360 | 51075 | 2285 | 4.28 |
| h2:0.3; snp.cau:0.01; ga:2 | 53370 | 51041 | 2329 | 4.36 |

5. Page 13 line 260: "Lloyd" not "Llyod". And this this presumably should be referred to as Reference 12?

*Response-5*: This has been corrected.

6. Page 17 line 333-336: this slightly assumes that these regions of 500 SNPs to the left and right of each causal SNP actually came up as "significant "(i.e exceeding threshold \alpha). What did you do if (in a given simulation replicate) a region did not meet this criterion?

*Response-6*: We defined regions around a causal SNP without an intermediate GWAS step to delineate regions. The described window (~500 SNPs on either side of the causal SNP) ensures SNPs within the region are jointly considered, accounting for linkage disequilibrium effects. In our simulation, we considered this region to be true causal. If a region did not meet the criterion (i.e. exceeding threshold \alpha), the region is considered as false negative.

7. Page 21 lines 426-431: In Figure 4 you show results for "Power", not Recall. How do you define Power? Or are you using the terms "Power" and "Recall" interchangeably? (Page 40 lines 792-795 suggests this may be the case).

*Response-7*: Thank you for pointing out this. This has been clarified within the revised manuscript.

8. Page 28 lines 569-585: I do not find this a very interesting summary of the results. Can you work a bit harder to summarise the results in Fig 4? Which methods are "best"? Does it matter that (for example) bR3 and bR2 have high F1score but very low Precision?

*Response-8*: Thank you for your feedback. Based on reviewers’ feedbacks, we have removed results from bR3 and focused only on the results from bR2. The overall F1 score for bR2 is higher compared to other fine-mapping models. However, its precision remains lower than that of other models. To simplify the discussion, we have analyzed this issue within the context of BayesR priors by comparing bR1 and bR2. Additionally, we have explained why this pattern is not observed with BayesC priors. In brief, we believe the lower precision is related to the default parameter settings for the metric π in the BayesR prior. This issue is discussed in detail in the "F1 Classification Score – Recall and Precision of the BLR Models" section (see lines 800–817).

Also, for comparisons between bR2 and external fine-mapping models, we focused on recall, incorporating a penalty for large credible set sizes that can lead to lower recall estimates. This topic, along with related considerations, is discussed in the "Comparison of the BLR Models to External Models" section (see lines 819–839).

9. Page 29 lines 587-595 (see also Page 39 lines 769-770). It is not really surprising that there are significant differences between the results for the different scenarios.

*Response-9*: Our methods were designed with specific performance expectations, and their behavior in the simulated scenarios aligns with these predictions. The results of these comparisons validate the accuracy of the simulation design and confirm that the methods perform as anticipated under the defined conditions.

***Reviewer #3***

Thank you for the comprehensive manuscript and extensive comparisons you have performed.

***Major comments***

1.) As you discussed in "Influence of parameters in simulations" it is difficult to disentangle under the setting of simulated parameters of each fine-mapped region. It would therefore be beneficial for comparisons of the methods if simulations parameters can set per genomic regions and xxx replicates are generated with the same parameters. A simple approach would be to use the list of identified T2D genes in Mahajan et al. Extract genotypes from UKB for a region around the genes and simulate phenotypes according to each combination of parameter values you want to evaluate.

*Response-1*: We agree that this is an important suggestion. Our study was designed to compare genome-wide fine-mapping BLR models (BayesC and BayesR) with region-specific fine-mapping approaches using BLR models (BayesR1/BayesR2 and BayesC1/BayesC2) and external fine-mapping methods (SuSiE/SuSiE-Inf and FINEMAP/FINEMAP-Inf). The rationale for this design is that genome-wide BLR models have demonstrated competitive predictive performance, and we aimed to evaluate their ability to fine-map. Our results suggest that genome-wide BLR models show comparable fine-mapping capabilities to state-of-the-art fine-mapping tools. This confirms that BLR models provide a unified framework for fine-mapping, parameter estimation, and prediction, offering a promising alternative for genome-wide analysis.

In the revised manuscript we have performed additional simulations and analyses to further support these findings.

2.) You are comparing fine-mapping methods under the assumption of one causal SNP per fine-mapping region. It is unclear why that assumption is necessary. In addition, it does not reflect the genomic architecture in T2D and other phenotypes (<https://www.nature.com/articles/s41588-018-0241-6#Fig3>). Therefore, it would be helpful for users of fine-mapping methods to see comparisons of the different methods when there is multiple causal signals presents in the each simulated region. This should be straightforward to implement in 1.) where you allow for xxx causal variants per simulated regions that jointly explain yyy% of phenotypic variance.

*Response-2*: Our simulation design considered only one causal variant per region. This assumption was emphasized because true positives, false positives, and false negatives were defined based on the identification of that single causal variant in the region. Additionally, this design aligns with the creation of credible sets similar to those described by Maller et al. (PMID: 23104008).

In the revised manuscript, we have performed additional simulations for scenarios with regions that contain two causal SNPs with varying degrees of LD between the two causal SNPs (please see Method section “Two causal genetic variants”: lines 182-189, Supplementary Table: S2 Table). Multiple credible sets were generated for all fine-mapping methods, and their ability to identify zero, one, or both causal SNPs was compared (please see description of results at lines 647-670).

3.) Although you have made an effort in "Credible sets for simulations" to explain how credible sets are defined, I have problems understanding exactly how they are defined. Could you please explain for a single fine-mapped region, how a credible set is defined and how many credible sets you obtained per that one fine-mapped region?

*Response-3*: For our analyses, two slightly different approaches were used to define credible sets (CSs) in the simulations and real data analyses, with the main difference being whether linkage disequilibrium was considered. For the simulations, we adopted the approach described by Maller et al. (PMID: 23104008), while for the real data analysis and an additional simulation, we utilized a definition aligned with the methodologies recently proposed by Zho et al. (PMID: 35853082) and Wu et al. (doi: 10.1101/2024.07.18.24310667). The approach described by Maller et al. (PMID: 23104008) is described in detail in Method section “Credible sets for simulation”: lines 403-411. The approach used for the real-data analysis and for the additional simulation is described in Method section “Credible sets for simulations with multiple causal SNPs”: lines 423-442, and Supplementary Text: S1Text, including a flowchart describing the process (see Supplementary Figure: S1Fig).

In the additional simulation analyses, we compared the outputs of these fine-mapping tools with our BLR methods, explicitly including both their default CS definitions and the CSs derived from the BLR methods. This dual comparison allowed us to assess not only the default performance of these tools but also how their results align with the CSs defined using our approach.

4.) When comparing different models, it would also be beneficial for the community to see a comparison based on PIPs. That is, using ROC curves an AUCs as has been done is previous benchmarks of fine-mapping methods.

*Response-4*: Thank you for your constructive feedback. In response to your suggestion, in the revised manuscript we have incorporated a comparison based on posterior inclusion probabilities (PIPs) using ROC curves and AUCs (please see description in Methods lines 444-452, and Supplementary Table: S3 Table). We believe this addition provides a more comprehensive evaluation of the models and allows for a clearer comparison of their performance in terms of variable prioritization. We believe this enhancement will be valuable for the community and further strengthen the rigor of our study.

***Minor comments***

1) Since you are allowing only for a single causal variant, it would make sense to run external fine-mapping methods such that they allow for exactly one causal variant.

*Response-1*: In our simulations, fine-mapping regions were defined around on a single causal variant. However, our BLR methods do not assume a single causal variant during fine mapping. Therefore, when comparing our methods to external fine-mapping approaches, it would be inappropriate to restrict the analysis to exactly one causal variant per region. We chose ten causal variants per region, consistent with the default setting in SuSIE-RSS.

2) It is unclear from the manuscript how PIPs are defined for BLR models.

*Response-2*: The posterior inclusion probability (PIP) of a genetic variant is defined as the proportion of iterations from the Gibbs sampling for which the genetic variant is included in the model with a non-zero effect (PMID: 29844615 and PMID: 37882742). This has been added to the revised manuscript (please see lines 276-278).

3) It is not evident how you BLR methods would deal with genotype missingness and varying sample size per variant when applied for fine-mapping.

*Response-3*: Missing genotypes were imputed using the mean imputation method, where the missing values for each genotype were replaced with the mean value calculated from the observed data for that genotype.

In the case of varying sample size per variant was accounted for in the reconstruction of $\boldsymbol{X}'\boldsymbol{X}$ and $\boldsymbol{X}'\boldsymbol{y}$ presented in equation in the diagonal elements of **D**:

$$\begin{matrix} \boldsymbol{X}'\boldsymbol{X} & =\boldsymbol{D}^{0.5}\boldsymbol{B}\boldsymbol{D}^{0.5}, \\ \boldsymbol{X}'\boldsymbol{y} & =\boldsymbol{D}\tilde{\boldsymbol{b}} \end{matrix}$$

where $D_{i}=\frac{1}{\sigma_{\tilde{b}_{i}}^{2}+\tilde{b}_{i}^{2}/n_{i}}$ if the genetic variants have been centered to mean 0, else $D_{i}=n_{i}$ if the genetic variants has been centered to mean 0 and scaled to variance of 1; thus, the diagonal elements of $\boldsymbol{D}$ represent the per variant sample size. The vector $\tilde{\boldsymbol{b}}$ is marginal SNP effects obtained from a standard GWAS, with $\sigma_{\tilde{b}_{i}}^{2}$ being the variance of the marginal effects from GWAS.

Thus, the number of observations per variant is incorporated into the reconstructed summary statistics. Furthermore, in a recent study (accepted for publication in *PLoS Genetics PGENETICS-D-24-00499R2 Improving polygenic prediction from summary data by learning patterns of effect sharing across multiple phenotypes*), we demonstrated that our BLR procedure is robust to varying degrees of missingness in the phenotypes.

This has been clarified in the revised manuscript (please see lines 253-268).
