# Supplementary material for: Enhanced genetic fine mapping accuracy with Bayesian Linear Regression models in diverse genetic architectures
Source: PLoS Genet. 2025 Jul 30;21(7):e1011783. doi: 10.1371/journal.pgen.1011783 (PMC12327644; doi:10.1371/journal.pgen.1011783)
Supplement: Attachment — Submitted filename: Response_to_reviewers_final.docx [file pgen.1011783.s030.docx]

**Dear editor,**

We thank the reviewers and editors for their thoughtful feedback, which has helped us to significantly improve the manuscript. In summary, we have made the following major revisions:

- **Revised title:** The manuscript title has been updated to *“Enhanced Genetic Fine Mapping Accuracy with Bayesian Linear Regression Models in Diverse Genetic Architectures”* to better reflect the scope and key contributions of our work.
- **Expanded simulation study:** We now include an in-depth evaluation of fine-mapping performance in regions with two causal variants, using varying sample sizes and multiple effect size configurations. This addition provides further insight into method robustness in more complex genetic architectures.
- **Improved fine-mapping analyses:** We have re-run the simulations to ensure correct initialization of prior parameters (e.g., π for BayesC and BayesR) and now report performance using similar coverage and purity metrics across all fine-mapping methods for a consistent and fair comparison.
- **Credible set comparison:** We added a detailed comparison of the credible set procedures used by each method, clarifying methodological differences and their impact on performance.
- **Additional figures:** Several new figures have been included to more clearly illustrate comparative results and support key findings.
- **Major text revisions:** The manuscript has undergone substantial revision to improve clarity, precision, and organization. Key sections, particularly the Introduction, Methods, Results, and Discussion, have been rewritten to enhance readability and address the reviewers’ concerns. All changes have been highlighted.
- **Supplementary Tables:** Tables S2–S5 contain the processed fine-mapping results for all methods across all simulations and can be used to reproduce the figures and results presented in this revised manuscript.

We believe these revisions have strengthened the manuscript considerably and we hope it will now meet the expectations of the reviewers and editorial team. Below in blue text, you will find our responses to the reviews’ comments.

***On behalf of all authors***

Peter Sørensen
Senior researcher,
Center for Quantitative Genetics and Genomics, Aarhus University

**Reviewer #1**

Thank you to the authors for addressing most of my previous comments. The revised manuscript is more comprehensive; however, I still have the following concerns:

**1. In this revision, the authors added a simulation comparison for two causal variants. In Table 4, they present a comparison of the credible set (CS) sizes, including the median size. However, what is LD between the two causal variants considered?**

We thank the reviewer for this question. To evaluate fine-mapping performance in regions with two causal variants, we extended our simulation study using imputed genotype data from the UK Biobank (chromosome 22; 89,458 SNPs post-QC). Quantitative traits were simulated across three sample sizes (200K, 250K, and 300K) and three effect size configurations: LL (two large effects), LS (one large and one small effect), and SS (two small effects). For each scenario, 100 replicates were generated using scaled genotypes and fixed residual variance.

Causal variants were randomly selected within a 2,001-SNP window (1,000 SNPs upstream and downstream from a randomly chosen index SNP), ensuring a broad distribution of pairwise LD values between the two causal variants. This setup was chosen to evaluate model performance under realistic and diverse LD structures.

We agree that a more systematic exploration of LD between closely linked causal variants is an important next step and could be addressed in future work. Related results are presented in Figure 5, Figures 6A–C, Figure S7, Figure S8, and the corresponding section of the revised manuscript.

**The authors showed the new CS definition reduced the number of variants in each CS. This conclusion is somewhat surprising to me. Could the author further analyze the results and provide a more detailed comparison between the new and previous CS definitions.**

We have clarified the definition and application of the credible set procedures in the revised manuscript. Specifically, the CS1 procedure is applied uniformly to all methods, while CS2 is used for the BLR models and the internal credible set procedures implemented in FINEMAP, SuSiE-RSS, and SuSiE-Inf. We also added a more detailed comparison of the credible set sizes across these procedures. Our results show that, on average, CS2 leads to slightly smaller credible sets compared to CS1 (see FigS2A-B and corresponding text in manuscript). We believe this is due to the additional LD-based filtering in CS2 (e.g., purity threshold of 0.5), which excludes variants with weak linkage to the lead SNP. However, the difference in average credible set size between CS1 and CS2 is modest. Notably, FINEMAP generally produced the largest credible sets across scenarios using the CS2 procedure. Across methods, average credible set sizes in the two-causal scenario were similar, ranging from 3.77 to 4.43 variants (Fig S8). FINEMAP had the largest sets (4.43), while SuSiE-Inf had the smallest (3.77). Other methods, including bR2, bC2, and SuSiE, showed minor differences, indicating comparable performance across models.

**2. Comment 1 also raises another question about the construction of CS. In the section "Credible sets for simulations with multiple causal SNPs", all variants are sorted by PIP and the CS are constructed based on the top sorted variants. At step 3, if a CS contains more than one SNP, the LD among these variants should be >0.5. In the additional simulations, if two causal variants have LD >0.5, the new CS will include both variants along with other highly correlated variants in the same CS at the construction part. It is unclear why the new CS construction results in a reduced number of SNPs. The authors should provide further clarification on this point.**

We agree that when two causal variants are in high LD (e.g., LD > 0.5), the CS2 procedure, based on PIP ranking combined with a pairwise LD threshold (purity ≥ 0.5), may include both causal variants and their correlated neighbors within the same credible set. However, the overall reduction in credible set size observed with CS2 is likely due to its additional filtering step, which explicitly removes variants with low LD to the lead variant (defined as the SNP with the highest PIP). This tends to exclude loosely linked variants that would otherwise be included in CS1, which relies solely on PIP ranking without considering LD structure.

Although CS2 may include multiple correlated causal variants, it is overall more restrictive, especially in scenarios with many low-PIP, low-LD variants. This leads to slightly smaller average credible sets, though the difference is modest (Fig S2A–B).

**Reviewer #2**

**This revised manuscript is improved by the addition of some simulations where the authors enforce a situation whereby there are multiple causal variants in a fine-mapped region. Unfortunately their description and discussion of the results obtained (lines 639-670 , page 30-31, S4 Table) seems to me quite opaque. The only thing that I find at all comprehensible are the AUCs mentioned in the text - but surely the full set of AUCs should be presented as a table (or supplementary table) and surely there should be some figures showing F1score, Precision and Recall? I do not understand the distinction the authors are making between CS>10 and CS <=10 and I really not understand what we are supposed to learn from the results shown in S4 Table and the text in lines 653-670. The authors need to do a much better job at guiding the reader through these results.**

We thank the reviewer for this helpful comment. In response, we have substantially revised the relevant section of the manuscript to clarify our presentation and interpretation of results from the two-causal variant simulations.

We evaluated the performance of fine-mapping methods under scenarios with two causal variants per region, across varying sample sizes (200k, 250k, 300k) and three effect size configurations (LL: two large effects, LS: one large and one small effect, SS: two small effects). The evaluation now include credible set procedures from FINEMAP, SuSiE-RSS, SuSiE-Inf, and the BLR models (which allow multiple credible sets).

As shown in Figure 5, F1 scores improved with increasing sample size across all methods. In the LL setting, bR1, bR2, bC2, and SuSiE-RSS performed best (F1 > 0.75 at n = 300k). Under LS, bR2 and bC2 maintained strong performance, while all methods performed poorly in the SS setting. However, BLR models generally outperformed SuSiE-Inf and FINEMAP under SS.

Ranking analyses (Fig S7A) showed that bC2 and bR2 had the best average F1-score ranks (2.5 and 2.7, respectively), followed by SuSiE-RSS and bR1. In contrast, FINEMAP, SuSiE-Inf, and bC1 ranked lowest (~5.0–5.4). While Recall, Precision, and AUC were generally similar across methods (Fig 6A–C), FINEMAP's lower F1-score stemmed from reduced Precision (Fig 6B, S7C).

To improve clarity, we have also added figures showing F1-score, Precision, and Recall, and expanded Supplementary Table S4 to include the full set of AUC values for all methods and scenarios.

Regarding the CS > 10 vs. CS ≤ 10 distinction: this threshold was used to qualitatively separate large and small credible sets to assess fine-mapping resolution. We have now clarified this in the revised text and simplified the comparison to better guide the reader.

Finally, average credible set sizes across methods were similar, ranging from 3.77 to 4.43 variants (Fig S8), with FINEMAP producing the largest and SuSiE-Inf the smallest. These results show that while performance varies by scenario, credible set sizes remain comparable across methods.

We hope the revised section and expanded figures/tables now provide a much clearer overview of these results.

**Apart from the simulations where there are multiple causal variants in a fine-mapped region, the authors seem to have chosen NOT to address my previous comment regarding including the "default" CSs that are actually output by FINEMAP, SuSIE-RSS and SuSIE-Inf. These are included in S4 Table (denoted by _a) but as far as I can tell they have NOT been included in any of the other tables or figures (e.g. S3 Table, Fig 4, S2 Fig, S3 Fig). I find this omission incomprehensible, seeing as I previously stated that I considered it ESSENTIAL that the authors include in their comparison the CSs that are actually output by FINEMAP, SuSIE-RSS and SuSIE-Inf, and, in their response, the authors stated "We fully agree that the default CS definitions are an integral aspect of these methods and a key feature that practitioners rely on."**

We appreciate the reviewer’s emphasis on including the default credible sets generated by FINEMAP, SuSiE-RSS, and SuSiE-Inf, and we agree that these are essential components of each method. We apologize for any confusion caused by how the results were previously presented.

In the revised manuscript, we have ensured that the default (internal) credible sets are clearly incorporated into the main analyses under the CS2 procedure. These internal credible sets are now consistently included in Figure 4, Supplementary Figures S2 and S3. We have also revised figure captions and the manuscript text to explicitly state when the internal procedures are used.

As shown in Figures 4 and S1, the use of CS2 generally results in lower Recall and substantially lower Precision compared to CS1, although the relative ranking of methods remains largely consistent. On average, CS2 produces slightly smaller credible sets, likely due to its LD-based filtering step (e.g., purity ≥ 0.5), which excludes weakly linked variants (Fig S2A–B).

We hope this clarification and the expanded presentation of results now fully address the reviewer’s concern.

**Reviewer #3**

**Major comments**

**You mention in the Author Summary that "that the BLR models better identify the genetic variants in terms of F1 score (recall and precision) and area under the receiver operating characteristic curve (AUC), in simulations."**

**However, in the results section you state that "Highest average AUC estimate was observed for FINEMAP-Inf (0.80) followed by SuSIE-Inf (0.79), and 2 (0.79). Highest 1 . score, averaged across all the twenty-four simulation (binary and quantitative) scenarios was observed for the BayesR region-wide model ( 2 ) [ 1 . score: 0.40] followed by SuSIE-Inf [ 1 . score: 0.35] and SuSIE-RSS [ 1 . score: 0.34] (Fig 1)."**

Thank you for pointing this out. We have revised the Author Summary and Results sections to ensure consistency and clarity regarding method performance.

Our updated rank-based evaluation confirms that bR2 consistently achieved the highest ranks in both F1-score and Recall across simulation settings, indicating robust and stable performance. Among the BLR models, we observed a consistent trend of bRgw < bR1 < bR2 for F1 and Recall, except under conditions of high polygenicity. A similar trend was also observed for the BayesC models.

Among the benchmarked methods, SuSiE-RSS performed well in terms of F1-score and Precision, while SuSiE-Inf achieved the highest Precision overall. FINEMAP showed moderate overall performance with variability in AUC. In contrast, FINEMAP-Inf ranked highest in AUC but performed poorly in F1-score and Recall, highlighting an imbalance across metrics.

bC1 ranked lowest overall, while bC2 and bR1 showed variable performance depending on the simulation scenario. Taken together, bR2 emerged as the most consistently strong method across F1 and Recall, with SuSiE-RSS and SuSiE-Inf also performing well depending on the metric considered.

**In addition, you state in "Comparison of fine-mapping models in “Two causal genetic variants”" that "We observed higher AUC estimates for BayesR ( 1: 0.82, 2: 0.85) compared to BayesC ( 1 and 2: 0.76). Highest AUC estimate was observed for FINEMAP and FINEMAP-Inf (0.90) followed by SuSIE-Inf (0.87), BayesR and SuSIE-RSS (0.79)."**

**How are these results compatible with your statements in the Author summary?**

Thank you for this comment. We have clarified the distinction in the revised manuscript. The Author Summary emphasizes that BLR models, particularly bR2, demonstrated consistently strong performance in terms of F1-score and Recall across simulation settings, reflecting a favorable balance between sensitivity and specificity in identifying causal variants.

In contrast, the AUC values reported in the two-causal variant simulation (now updated) reflect each model’s ability to prioritize causal SNPs based on PIP rankings, not necessarily the precision of the final credible set. While FINEMAP and SuSiE-Inf achieved higher AUCs, they often produced larger credible sets, which lowered their Precision and, in turn, F1-score.

The updated results reinforce this interpretation: bR2 remains among the top performers in F1-score, while FINEMAP and SuSiE-Inf excel in AUC. We have revised both the Author Summary and Results sections to ensure consistency and have clearly stated how different metrics highlight complementary aspects of method performance.

**Minor comments**

**Would it be possible to address whether F1 score or AUC is more suitable for comparing methods?**

We used both **F1-score** and **AUC** to evaluate fine-mapping methods, as they capture different aspects of performance. Since the primary goal of fine-mapping is to accurately identify true causal variants, **F1-score**—which balances **precision** (the proportion of predicted positives that are true positives) and **recall** (the proportion of true positives that are identified)—is particularly well-suited for this purpose.

In contrast, **AUC**, calculated from posterior inclusion probabilities (PIPs), reflects a model’s ability to **rank** causal variants higher than non-causal ones. This metric is valuable in simulations, where the true causal SNPs are known, but it does not directly evaluate credible set performance.

For real data, where causal variants are unknown, we use **AUC/R²** as a proxy for overall model fit. While it provides only an indirect measure of fine-mapping accuracy, it captures a model’s capacity to explain the genetic signal and differentiate between more and less likely causal variants without requiring ground truth.

**The "Application in simulations" has become a bit unorganized and hard to follow. Would it be possible to make the results more concise in order to crystalize whether BLR methods improve fine-mapping over existing methods?**

We agree that the “Application in simulations” section needed better organization and clarity. We have rewritten this section to improve flow and conciseness, and to better highlight the comparative performance of the BLR methods. Additionally, we have added a new simulation study involving two causal variants to further support our evaluation and clarify the advantages of the BLR models over existing fine-mapping methods.
